# Affordable Biocidal Ultraviolet Cured Cuprous Oxide Filled Vat Photopolymerization Resin Nanocomposites with Enhanced Mechanical Properties

**DOI:** 10.3390/biomimetics7010012

**Published:** 2022-01-10

**Authors:** Markos Petousis, Nectarios Vidakis, Emmanuel Velidakis, John D. Kechagias, Constantine N. David, Stefanos Papadakis, Nikolaos Mountakis

**Affiliations:** 1 Mechanical Engineering Department, Hellenic Mediterranean University, Estavromenos, 71410 Heraklion, Greece; vidakis@hmu.gr (N.V.); m.velidakis@hmu.gr (E.V.); mh90@edu.hmu.gr (N.M.); 2 General Department, University of Thessaly, 41500 Larissa, Greece; jkechag@uth.gr; 3 Manufacturing Technology & Production Systems Laboratory, School of Engineering, International Hellenic University, Serres Campus, 62124 Serres, Greece; david@ihu.gr; 4 Biology Department, University of Crete, Voutes University Campus, P.O. Box 2208, 70013 Heraklion, Greece; stefpap@biology.uoc.gr

**Keywords:** stereolithography (SLA), 3D printing, antibacterial, additive manufacturing (AM), Cuprous Oxide, resin, mechanical, nanocomposites

## Abstract

In this study, Cuprous Oxide (Cu_2_O), known for its mechanism against bacteria, was used as filler to induce biocidal properties on a common commercial resin stereolithography (SLA) 3D printing resin. The aim was to develop nanocomposites suitable for the SLA process with a low-cost process that mimic host defense peptides (HDPs). Such materials have a huge economic and societal influence on the global technological war on illness and exploiting 3D printing characteristics is an additional asset for these materials. Their mechanical performance was also investigated with tensile, flexural, Charpy’s impact, and Vickers microhardness tests. Morphological analysis was performed through scanning electron microscopy (SEM), atomic force microscopy (AFM), and energy-dispersive X-ray spectroscopy (EDS) analysis, while the thermal behavior was studied through Thermogravimetric Analysis (TGA). The antibacterial activity of the fabricated nanocomposites was investigated using a screening agar well diffusion method, for a gram-negative and a gram-positive bacterium. Three-dimensional printed nanocomposites exhibited antibacterial performance in all loadings studied, while their mechanical enhancement was approximately 20% even at low filler loadings, revealing a multi-functional performance and a potential of Cuprous Oxide implementation in SLA resin matrices for engineering and medical applications.

## 1. Introduction

The discovery of the antibiotic penicillin in 1928 significantly transformed medicine, while the prolonged use of antibiotics ever since has necessitated the development of other treatments, with antibiotic-resistant bacteria affecting at least 2 million people each year, according to the Centers for Disease Control and Prevention (CDC) [1]. Microbial contamination of air, water, and soil by various microorganisms causes issues in living situations, as well as in public health and industry. As a result, antibiotic resistance genes have become more common in numerous bacterial species, including humans and animals [2]. The immunity system has mechanisms to inhibit or kill bacteria through the host defense peptide (HDP) and their physicochemical characteristics are imitated by synthetic (co)polymers [3], while metals, such as copper, silver, titanium, and zinc have characteristics that inhibit the growth of different bacteria, with their antibacterial efficacy verified in technologically appealing materials in fabrics, paints, and coatings that incorporate copper-based active granules or pigments [2]. Additionally, metal Nanoparticles (NPs) are the most promising in this field since they have exhibited significant antibacterial properties over the last few decades and have become more widely used in industry [4]. Different metallic and metal oxide nanoparticles (NPs) may have very promising and effective roles as antibacterial agents due to their enormous surface-to-volume ratio and crystalline shape, which elicit biological reactions that differ from those elicited by metals in their typical ionic form [5]. Still, the mechanisms underlying metallic nanostructures’ biocidal action are not fully understood [5]. Physical contact between nanomaterials and bacteria, such as van der Waals interactions, electrostatic interactions, hydrophobic interactions, and receptor-ligand interactions, can result in membrane destruction, the inactivation of important cellular components, and bacteria death. Bacteria are classified into two groups based on the structure of their cell walls: gram-negative and gram-positive. Gram-negative bacteria have a thinner peptidoglycan layer than gram-positive bacteria because they have a unique outer membrane [6]. In this work, a gram-negative and a gram-positive bacterium were studied.

Over the last few decades, additive manufacturing (AM) has gained continuously increasing interest either at the academic or engineering level [7,8]. AM techniques are the most promising manufacturing methods for the industrial sectors in the future [9]. Applications have already been implemented in the automotive [10,11,12], electronics [13,14,15,16], packaging [17,18,19,20], medical, [21,22,23,24], and other economic sectors. Existing AM technologies have differences not only in their operating principles but also in the materials utilized in each method [25,26]. Apart from these differences, all AM techniques share the same layer-by-layer manufacturing principle [27]. Commercially known AM technologies are fused filament fabrication (FFF), selective laser sintering (SLS), and stereolithography (SLA) [28]. FFF has been widely utilized even for home usage [27,29]. Consequently, sufficient research has been conducted on the materials of this AM technique, providing a continuously widening variety of composite materials with enhanced mechanical [30,31,32,33,34], thermal, [35,36,37], and other properties. In SLS, a laser beam melts the raw material, which is a powder form. The process has great ability to make end products for most industrial applications due to its build accuracy, high productivity for customized and complex-shaped things, and strong mechanical qualities [38]. For this reason, it has been used in several industrial and scientific fields, from prototypes to the fabrication of spare parts and small series in automotive and aerospace fields, to customized prostheses for biomedical applications [39]. SLA is an AM technique based on the photosensitivity of utilized materials [40], which are met in liquid form and are most known as ultraviolet (UV)-cured resins [41]. Liquid resins are poured in a transparent bottom tank, and a light source with a spot on the micro-scale is electronically driven to initiate the curing of the resin [42]. This process is repeated, and the final 3D printed part emerges on the build platform of the 3D printer. The light source type is the fundamental difference between SLA, digital light processing (DLP), and liquid crystal display (LCD) technologies [43]. The latter method has driven the price of UV resin 3D printers to low levels, consequently making them a competitor to the FFF process for everyday utilization [44].

Even enough studies have been conducted in the general field of resin composites [24,45], nanotechnology and especially the utilization of nanoparticles could provide a potential in photosensitive resin nanocomposites [46]. The SLA 3D printing technology usually produces parts with small layer heights. The most common layer height setup is 50 microns, and it is not prohibited to further lower the layer height to 25 microns or less [47]. Such small gaps between the build platform and the tank’s bottom create difficulties in the 3D printing process, usually due to the high viscosity of the materials used and the “cups” effect [47,48]. Such problems have great potential to be resolved through the utilization of nanotechnology. Nanoscale size in combination with the high transferability of properties through fine particle dispersion in the matrix do not create big changes in the viscosity of the nanocomposite, and as a result, 3D printing does not require special procedures [40,49].

The recent Covid-19 pandemic situation has aroused the need for 3D printing utilization and its fully operational components [50]. Difficulties created in logistics and production rates were effortlessly resolved by enabling the 3D printing community worldwide [51,52]. Most known case studies involve the manufacturing of face shields and components of the respiratory valve machine [53,54,55]. Even though the main AM method utilized in these projects was FFF, SLA could provide optimized solidarity in parts and faster turnover times [56]. Medical applications require the utilization of materials with good mechanical performance and antibacterial properties [23,57], except for thermal stability. Materials with biomimetic characteristics have the potential to be used in a variety of applications and therapeutic delivery strategies to tackle antibiotic-resistant bacteria infection [1], while SLA already has sufficient implementation in the dental and wider medical sector; nevertheless, the properties of the materials used are not at the optimized level.

This work aimed to develop materials in a form suitable for SLA 3D printing, mimicking HDPs functionalities, to be suitable for use in corresponding engineering and medical applications. To achieve this, metals’ biocidal properties were exploited. More specifically, a common SLA resin was utilized as a matrix material filled with Cuprous Oxide (Cu_2_O) at different loadings. Although Copper’s mechanism of cell death is not yet fully known, Copper oxides (CuO and Cu_2_O) have a contact killing mechanism that causes significant damage to the cell membrane in bacteria [58]. Copper nanoparticles’ antibacterial properties were attributed mostly to their ability to adhere to bacteria due to their opposite electrical charges, culminating in a reduction reaction at the bacterial cell wall [2]. The process for the preparation of the nanocomposites was implemented with common low-cost laboratory equipment. No similar research has been presented in the literature so far. Cu_2_O material antibacterial properties are thoroughly analyzed in literature [59], while the implementation of Cuprous Oxide nanoparticles in SLA resin matrices has not yet been studied, according to the authors’ knowledge. The mechanical performance of the developed nanocomposites was also studied, and morphological and thermal analyses were performed to determine the effectiveness of the process. The antibacterial properties were analyzed using the screening method of agar well diffusion. The results showed that the implementation of nanoparticles in commercially utilized SLA resins has great potential, without the need for high-end technology equipment, since the nanocomposites developed exhibited antibacterial performance while having, at the same time, enhanced mechanical response.

## 2. Materials and Methods

### 2.1. Materials

For this study, a widely utilized commercially available resin was selected as the matrix material. Specifically, Formlabs Standard Clear V4 (Formlabs Ohio Inc., Millbury, OH, USA) was procured from a local supplier. Standard Clear V4 resin (SC), according to the manufacturer’s safety data sheet, consists of 55–75% of urethane dimethacrylate, 15–25% methacrylate monomer(s), and less than 0.9% diphenyl (2,4,6-trimethybenzoyl) phosphine oxide. Such a composition of monomers enables the resin’s photoreactive behavior for utilization in the stereolithography (SLA) AM technique. As a filler, an antibacterial reactor agent utilizing nanoparticles of Cuprous Oxide I (Cu_2_O) was used. Cu_2_O nanopowder was procured from Nanografi (Nanografi Inc., Ankara, Turkey). It features an average particle size of 80 nm. The nanopowder purity is 99.5%, while the melting point of the material is 1.240 °C, according to the supplier’s datasheet.

### 2.2. Nanocomposites and Specimens Fabrication

**Figure 1:**
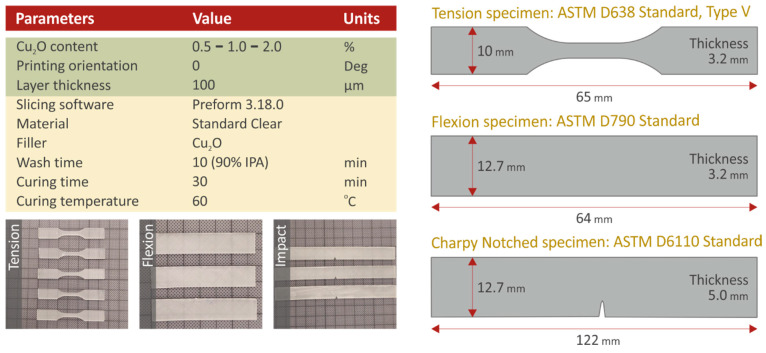
Fundamental SLA 3D printing settings, pictures, and sketches of the 3D printed specimens prepared in this work. Specimens were manufactured according to the corresponding American Society for Testing and Materials (ASTM) International Standard for each different type of test.

A high shear force mixer was utilized for the mixing procedure. SC resin was weighed and mixed with nanoparticles for filling ratios of 1.0 wt.% and 2.0 wt.% The mixing duration was 30 min to ensure the optimized dispersion of nanoparticles in the nanocomposites. Before the pure resin entered the 3D printer tank, a degasification procedure was conducted using a lab vacuum chamber at a room temperature of 21 °C. The 3D printer utilized for the SLA process was a Formlabs Form 2 (Formlabs Ohio Inc., Ohio, USA), equipped with a resin tank of Formlabs Tank LT. Form 2 was equipped with a laser light source with a wavelength of 450 nm and a laser spot of 150 µm. Slicing and gcode export were conducted using PreForm software version 3.16 (Formlabs Ohio Inc., Ohio, USA), and the layer height was fitted at 100 microns. All specimens were oriented with their largest surface on the build platform. After the 3D printing process, specimens were removed carefully from the build platform, and they were instantly emplaced in the Formlabs Form Wash machine. In this machine, they were washed in an isopropyl alcohol (IPA) solution of 90% purity. The washing time was set according to the Formlabs specification at 10 min. Afterward, the specimens were dried under room conditions (22 °C, 50% RH). The final UV curing process was performed using a Formlabs Form Cure (Formlabs Ohio Inc., Ohio, USA) machine. Final curing was conducted for 30 min at 60 °C. Figure 1 presents the fundamental 3D printing setting.

### 2.3. Mechanical Performance Testing

**Figure 2:**
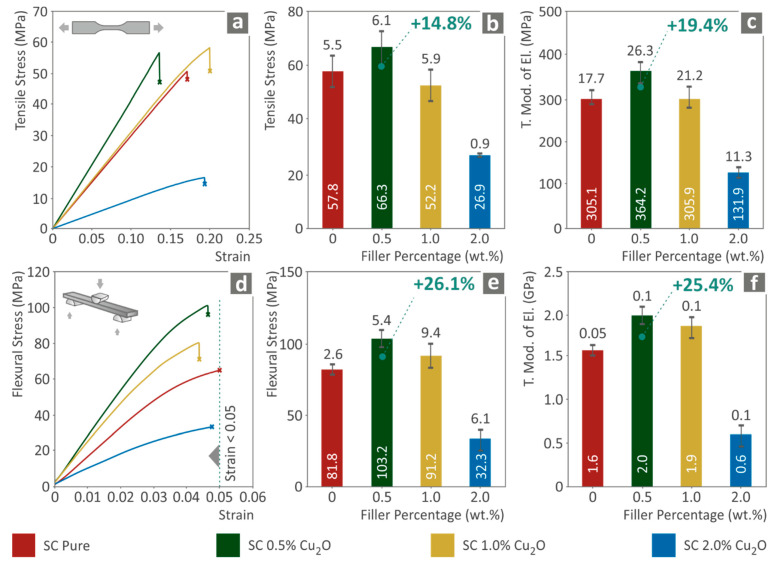
(**a**) Typical tensile stress (MPa) to strain (mm/mm) curve, (**b**) Tensile stress at break (MPa) to filler loading (wt.%), (**c**) tensile elastic modulus (MPa) to filler ratio (wt.%), (**d**) Typical flexural stress (MPa) to strain (mm/mm) curve, (**e**) Flexural stress at break (MPa) or 5.0% strain -if no break existed- to filler loading (wt.%), (**f**) flexural modulus of elasticity (MPa) to filler ratio (wt.%).

The mechanical properties were analyzed with tensile, flexural, impact, and Vickers microhardness testing. Tensile tests were conducted according to the ASTM D638-02a international standard on five (5) type V specimens of 3.2 mm thickness. The equipment used for the tensile tests was an Imada MX2 (Imada Inc., Northbrook, IL, USA) machine equipped with standardized grips. The tension speed was set to 10 mm/min while the tests were conducted in room conditions (21 °C, 50% RH). An Imada MX2 machine was also utilized for the flexural tests. The flexural setup was established in the machine according to the ASTM D790-10 international standard. Five specimens of 3.2 mm thickness and geometry as specified in Figure 2 were tested in three-point bending tests with the chuck speed set at 10 mm/min. The ASTM D6110-04 international standard was followed for Charpy’s notched specimens impact measurements. Five specimens were tested for their impact strength using a Terco MT220 machine (Terco AB, Huddinge, Sweden). Randomly selected specimens were utilized for Vickers microhardness measurements according to the ASTM E384-17 international standard, which was followed for the five measurements conducted on each case of the fabricated nanocomposite material. Microhardness measurements were taken since they are directly related to the material’s mechanical response [60].

### 2.4. Morphological, Thermal, and Antibacterial Analysis

Scanning electron microscopy (SEM) images were acquired at different magnification levels from the fractured surface of the tensile specimens. A JEOL 6362LV (Jeol Ltd., Norwood MA, USA) apparatus was utilized for this purpose. Samples were randomly selected and sputter-coated with gold (Au) to avoid charging effects. The electron microscope was set in high vacuum mode at 20 kV acceleration voltage. Energy-dispersive X-ray spectroscopy (EDS) analysis was also conducted on the same electronic microscope on non-coated samples from each material to verify the elements in each case.

Micro-scale analysis was conducted on the surface topology of the cured specimens using the atomic force microscopy (AFM) technique with a Microscope Solver P47H Pro (NT-MDT, Moscow, Russia) apparatus. Commercially available silicon cantilevers with a scanning frequency of 1 Hz, cantilever spring constant of 35 N/m, tip cone angle of 20°, and tip radius of 10 nm were utilized at a resonant frequency of 300 kHz. A part of the 3D printed specimens was also utilized for Thermogravimetric Analysis (TGA) measurements in samples of approximately 10 mg. A Perkin Elmer Diamond TGA/DTGA (Perkin Elmer Inc., Waltham, MA, USA) apparatus was used, and the temperature was measured from 40 °C to 550 °C with a ramp of 10 °C/min.

The antibacterial performance of the developed nanocomposites was determined using the agar well diffusion method in a microbiological lab for two (2) different bacteria. Gram-negative *Escherichia coli* (*E. coli*) and gram-positive *Staphylococcus aureus* (*S. aureus*) bacteria were cultivated in Petri dishes of 85 mm diameter. Each bacterium was cultivated with a specific growth material in different Petri dishes. Specimens of 12.7 mm diameter and height of 5.00 mm were placed in each petri dish. Petri dishes were placed in an oven at 37 °C for a period of 24 h targeting the optimized diffusion of antimicrobial agents in the agar and inhibiting germination and growth of the test microorganism. Subsequently, the inhibition zones (IZ) of 3D printed specimens were measured peripherally using optical equipment.

## 3. Results

### 3.1. Mechanical Performance Analysis

Figure 2a–c present the tensile testing results for the fabricated nanocomposites compared to the clear resin utilized as matrix material. It is found that the presence of Cuprous Oxide in the nanocomposite affects the tensile behavior of the matrix material. Maximum enhancement was measured in the case of SC Cu_2_O 0.5 wt.% nanocomposite, with the tensile stress at break measured to be approximately 15.0% higher than neat SC (Figure 2b), and the corresponding tensile modulus of elasticity was increased approximately 20.0% (Figure 2c). Higher filler loadings exhibited degraded performance, which was attributed to increased Cu_2_O presence in the nanocomposites and a plausible difficulty in photopolymerization, resulting in not completely photopolymerized specimens.

Figure 2d–f present the corresponding results from the flexural tests conducted on the specimens. A similar tensile behavior trend was observed in the flexural stress analysis. A stiffening effect was observed for all fabricated nanocomposites, which was a result of the introduction of metal oxides into the materials. The flexural stress at break and flexural modulus of elasticity were measured enhanced by approximately 25.0% (Figure 2e) compared to neat SC resin. Deterioration over 0.5 wt.% filler ratio was also presented. Such performance is attributed to the increased filler loading as described above.

**Figure 3:**
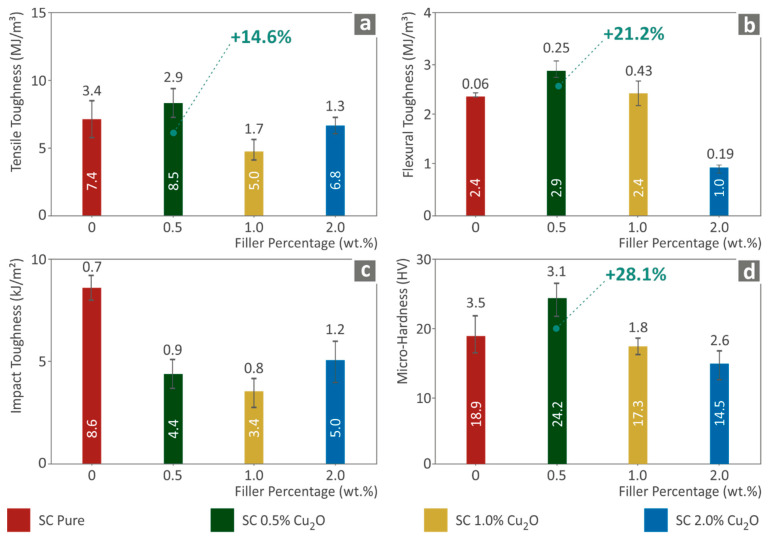
(**a**) Average tensile toughness (MJ/m^3^) to filler loading (wt.%), (**b**) Average flexural toughness (MJ/m^3^) to filler ratios (wt.%), (**c**) Charpy’s notched impact toughness (kJ/m^2^) to filler loadings (wt.%), (**d**) Vickers microhardness (HV) to filler ratios (wt.%).

In Figure 3, a comparative analysis is depicted for the tensile and the flexural toughness (MJ/m^3^), i.e., the integral of the corresponding stress-strain curves of all tested specimens. This analysis is related to the absorbed energy from each trial until breakage occurred or 5.0% strain for flexural tests when no break occurred, following the corresponding standard instructions. Based on the results presented previously, the calculated toughness for tensile and flexural tests exhibits a similar performance for the studied nanocomposites. Low filling ratios enable the potential to create plausible stronger interface bonds between photopolymerized polymeric chains of SC resin with filler nanoparticles. Such interface fusion enhances the stiffness of the material, followed by an increase in the measured stresses and the ability to absorb more energy during deformation. The mechanism that creates obstacles in higher filling loadings in nanocomposites will be further analyzed in the Section 4.

Finally, impact toughness and Vickers microhardness tests results are shown in Figure 3c. Impact tests did not follow the same trend as the remaining mechanical tests, exhibiting an intense degradation in the ability to absorb impact energy. Generally, SLA-utilized resins perform in a non-ductile manner. This high stiffening behavior worsens the ability to enhance the impact property. In addition, metal oxides are also materials with low to almost non-existent ductility, which results in further enhancement of stiffness, as shown above. Vickers microhardness follows the already presented performance of the nanocomposites (Figure 3d). Following the quasi-static tests, the specimen surface behaved accordingly to the internal structure performance.

### 3.2. Thermal, Morphological, and Antibacterial Analysis

#### 3.2.1. Thermal Analysis

**Figure 4:**
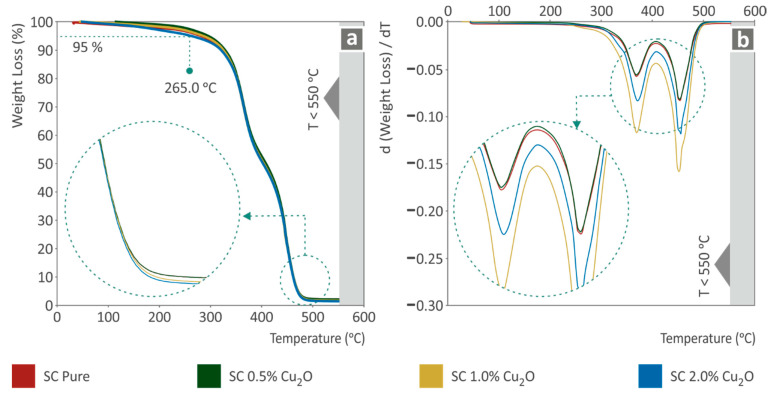
(**a**) Sample’s weight (%) to temperature (°C), (**b**) weight loss rate (mg/mg) to temperature (°C).

Thermogravimetric analysis was conducted on samples acquired from 3D printed specimens. The results are shown in Figure 4. In Figure 4a, it can be observed that the addition of filler to each nanocomposite is in agreement with the weight, as the remnants agree with the corresponding loading. It should be mentioned that even pure SC resin had not completely burned at the highest achieved temperature of 550 °C, and as a result, the existence of the filler in the nanocomposite is a qualitative assumption and not an exact measured one. In Figure 4b, in which the weight loss rate is presented, it is shown that the addition of Cu_2_O over 0.5 wt.% in the nanocomposite is intensively changing the thermal performance of the fabricated nanocomposite materials. Thus, such an effect enhances the mechanical deterioration behavior, as the thermal degradation of 1.0 wt.% and 2.0 wt.% nanocomposites is plausibly attributed to a not totally, or bad-quality photopolymerization procedure, as for the filler existence in the nanocomposite.

#### 3.2.2. Morphological Analysis

**Figure 5:**
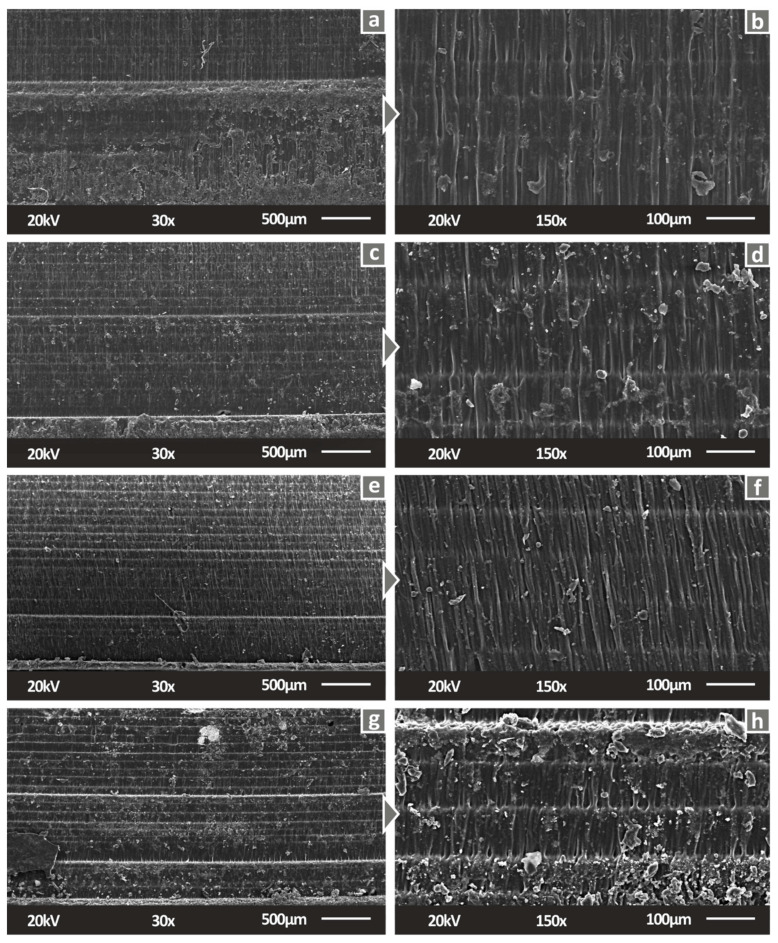
Side surface of tensile specimens in 30× magnification for (**a**) Pure SC, (**c**) SC Cu_2_O 0.5 wt.%, (**e**) SC Cu_2_O 1.0 wt.%, (**g**) SC Cu_2_O 2.0 wt.%, same surfaces in 150× magnification for (**b**) Pure SC, (**d**) SC Cu_2_O 0.5 wt.%, (**f**) SC Cu_2_O 1.0 wt.%, (**h**) SC Cu_2_O 2.0 wt.%.

For a complete view of this study’s process, morphology analysis was conducted utilizing SEM on the side and fractal surface of tensile specimens. Figure 5 present the side surfaces of the specimens at two magnification levels. The side surface of the SC resin specimen exhibits a smooth morphology; the layering is still visible and is in coherence with the 3D printing settings. The fusion quality of the layers is observed to be good. A similar morphology is observed for the nanocomposite SC Cu_2_O 0.5 wt.%, while for the higher filler’s concentration, nanocomposite fusion quality shows degradation. Additionally, the implications of plausible agglomerations are shown for these nanocomposites. These observations enhance the assumption that there were difficulties in the photopolymerization process for the nanocomposites with 1.0 wt.% and 2.0 wt.% loadings.

**Figure 6:**
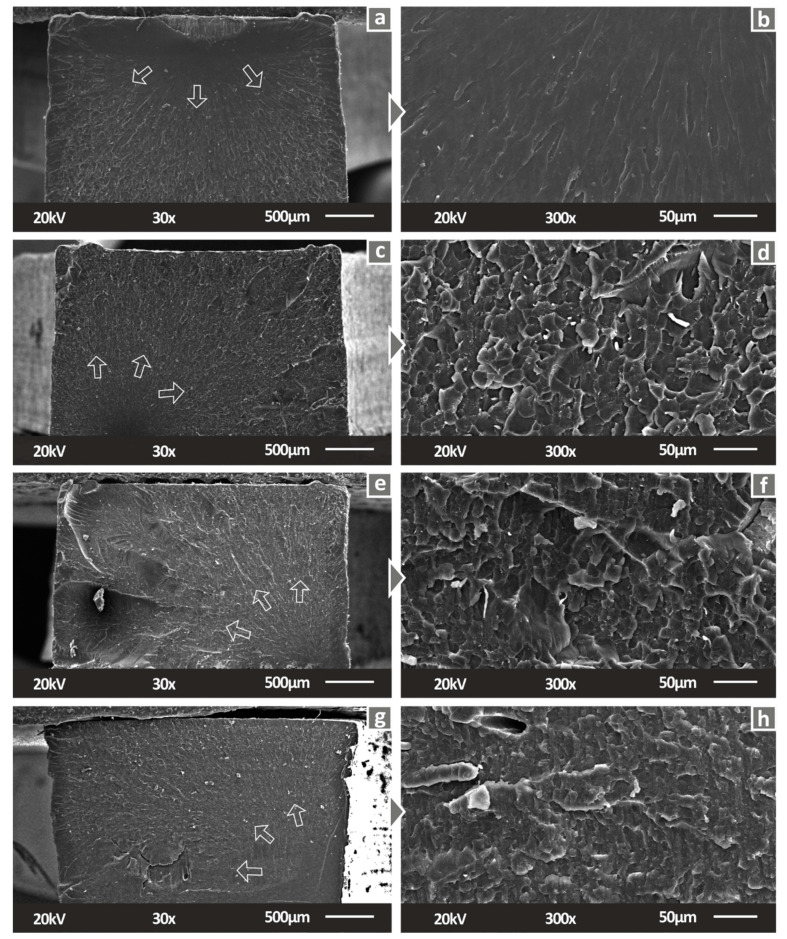
Fracture surface of tensile specimens in 30× magnification for (**a**) Pure SC, (**c**) SC Cu_2_O 0.5 wt.%, (**e**) SC Cu_2_O 1.0 wt.%, (**g**) SC Cu_2_O 2.0 wt.%, same surfaces in 300× magnification for (**b**) Pure SC, (**d**) SC Cu_2_O 0.5 wt.%, (**f**) SC Cu_2_O 1.0 wt.%, (**h**) SC Cu_2_O 2.0 wt.%.

Figure 6 presents SEM images from the fracture area of the tensile specimens. Following the side surface analysis, the fracture area provides more information and proof of the deteriorated processing at higher filler ratios in the fabricated nanocomposites. Plausible agglomerations are visible, while the total morphology of the fractal area in the two higher filled nanocomposites exhibited slight voids, which probably occurred due to the pure photopolymerization of the matrix SC resin. The fracture area morphology also exhibits a stiffening effect due to the introduction of Cuprous Oxide in the nanocomposites. The traveling direction of the break that occurred during the tensile test is clear in the case of pure SC and SC Cu_2_O 0.5 wt.%. A slight decrease in the ductility of the resin was observed even at 0.5 wt.% nanocomposites. The brittleness effect occurred due to the metal oxide presence, which is visible in the fractal areas of higher filler loadings images.

**Figure 7:**
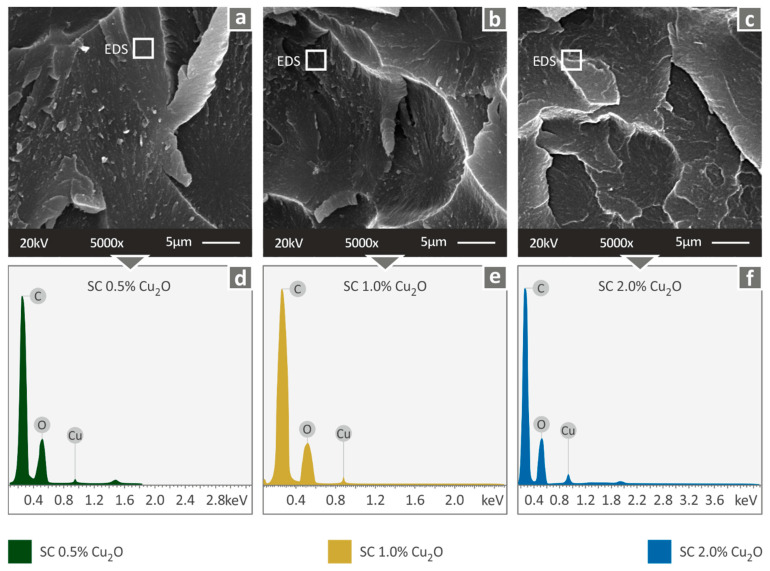
Fracture area high magnification captures at 5000× level and the corresponding EDS analysis results: (**a**) SC Cu_2_O 0.5 wt.% SEM 5000×, (**b**) SC Cu_2_O 1.0 wt.% SEM 5000× (**c**) SC Cu_2_O 2.0 wt.% SEM 5000× (**d**) SC Cu_2_O 0.5 wt.% EDS graph (**e**) SC Cu_2_O 1.0 wt.% EDS graph, (**f**) SC Cu_2_O 2.0 wt.% EDS graph.

Higher magnification captures were taken in the fractal area and EDS analysis on the specimens’ surface. Figure 7 presents images and EDS results for the nanocomposites fabricated for the current study. The presence of Cu in the EDS graphs, because of the addition of Cuprous Oxide, is expected in each fabricated nanocomposite, with the peaks verifying the good dispersion of the filler in all cases.

Finally, the morphology and the quality of the procedure were also evaluated with atomic force microscopy (AFM) analysis on the 3D printed specimens’ surfaces of all tested materials. The surface topology of higher filled nanocomposites presents a vigorous roughness increase, plausibly attributed to slight agglomerations created. The SC Cu_2_O 0.5 wt.% nanocomposite’s surface roughness has a fine quality finishing, with low differences, implying that nanoparticles had dispersed fine, and the polymerization process faced no difficulties.

#### 3.2.3. Nanocomposites Biocidal Performance

**Figure 8:**
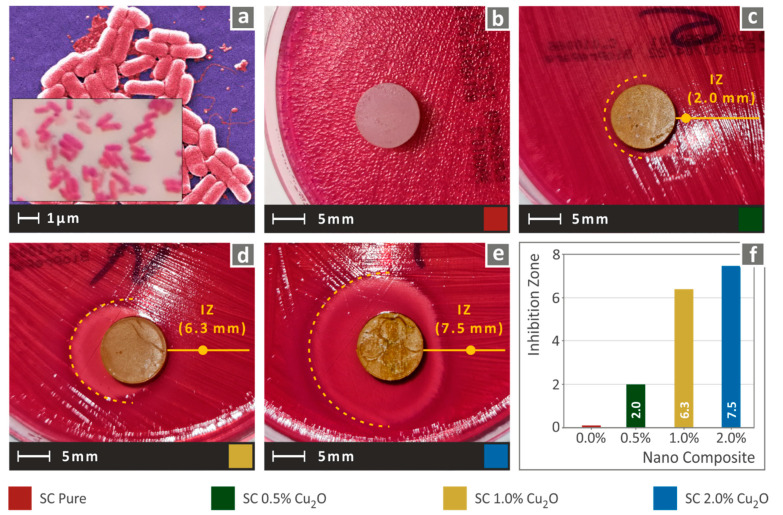
(**a**) typical *E. Coli* morphology, (**b**–**e**) Vertical captures after 24 h cultivation of tested specimen in a petri dish for each corresponding tested material, (**f**) Comparative graph of the measured inhibition zones to filler loading (wt.%).

To mimic HDPs functionalities in the nanocomposites developed, biocidal characteristics must be induced, and to achieve that, the Cuprous Oxide filler’s properties were exploited in the study. The antibacterial performance of the prepared nanocomposites was investigated using the agar well diffusion method, and the results for the two tested bacteria are presented in the following figures. Images after 24 h of cultivation in Petri dishes of gram-negative *E. coli* are displayed in Figure 8. The antibacterial properties of Cuprous Oxide were introduced in the fabricated nanocomposites. In particular, the increase in Cuprous Oxide concentration in the matrix increased in the inhibition zone. It should be mentioned that the SC resin did not exhibit any antibacterial performance, as expected, which creates a great potential for Cuprous Oxide addition in resin matrices.

**Figure 9:**
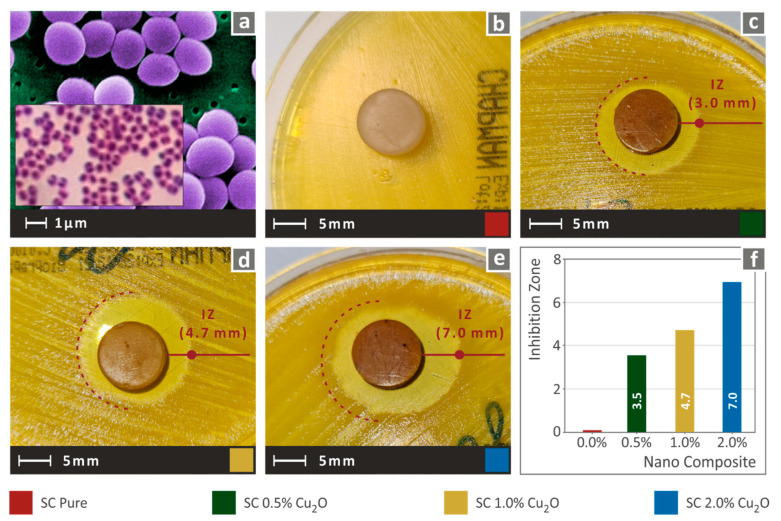
(**a**) typical *S. aureus* morphology, (**b**–**e**) Vertical captures after 24 h cultivation of tested specimen in a petri dish for each corresponding tested material, (**f**) Comparative graph of the measured inhibition zones to filler loading (wt.%).

Similar behavior was observed in the case of the gram-positive *S. aureus* testing case. As shown in Figure 9, antibacterial activity was introduced in the nanocomposites by the addition of Cu_2_O as a filler in the SC resin. Indeed, in the case of 0.5 wt.% filler ratio, the inhibition zone against *S. Aureus* was further increased compared to the corresponding *E. Coli*. Following a similar trend to the *E. coli* bacterium behavior, an increase in the Cuprous Oxide presence increased the inhibition zone to *S. aureus*. As a result, the optimization process of the antibacterial behavior is only impeded by the processability of the materials, which is mainly focused on the effective photopolymerization and the reduction of agglomeration effects.

## 4. Discussion

In this work affordable materials suitable for SLA 3D printing, mimicking HDPs performance were prepared and fully characterized. Such materials with antibacterial performance for medical purposes are developed for the design of medical tools, antibacterial surfaces, etc., that prevent bacteria germination and growth. These materials, in most cases, are not designed for prolonged contact with living organisms or to be introduced in the human body since malicious tissue interaction with the composite or other side effects that affect the cells are possible. For this reason, cytotoxicity studies are also required in these cases, and the safety of materials for in vivo use should be ensured. Studying the in vivo antibacterial efficacy of the developed materials of the study was not in the scopes of the study.

The purpose of this work was to introduce antibacterial properties to an SLA resin by employing a low-cost antibacterial filler and common laboratory tools. The principle of the process followed for the preparation of the nanocomposites is based on high-shear forces for the mixing process of the filler with the matrix material. Mechanical, morphological, thermal, and antibacterial analyses were conducted on SLA 3D printed specimens of SC resin and Cuprous Oxide-filled nanocomposites. Mechanical analysis results indicated that the addition of Cu_2_O at a low filling ratio enhanced the tensile and flexural performance of the SC resin. A higher filler concentration exhibited a sudden degraded mechanical performance, as the fabricated SC Cu_2_O 1.0 wt.% and 2.0 wt.% nanocomposites performed over 50% lower than the lower filler loading both in tensile and in flexural stress tests. The SLA 3D printing process induces a laser beam in a specific laser spot (150 microns to the utilized 3D printer) to initiate the polymerization process to the photosensitive resin. Nanoparticles induced in the SC resin matrix could create diffusion effects, especially in the case of high concentrations, where the agglomeration effect could plausibly occur at the higher filler loadings. Low filler addition enables nanoparticles to disperse in the matrix most finely, reducing even the possibility of slight agglomeration. The 0.5 wt.% loading exhibited overall the more enhanced mechanical response, with the tensile strength being 26.1% higher than the matrix material, the tensile elastic modulus 25.4% higher, indicating a stiffer behavior of the material with the addition of the filler, flexural strength was increased 14.6%, and flexural modulus of elasticity was also 21.2% higher than the matrix material, verifying the stiffer behavior of the nanocomposite in this test. Even at this low filler loading, the nanocomposite exhibited antibacterial performance for the two bacteria studied, showing potential for multi-functional performance in this nanocomposite. The process followed, employing common laboratory equipment and a rather low-cost filler with antibacterial properties, introduced a nanocomposite with low filler loading of 0.5 wt.% and optimum performance, which has obvious advantages. Apart from the improved mechanical performance and the antibacterial response, low filler loading is easier to process, and the overall cost for the preparation of the nanocomposite is not significantly increased when compared to the cost of the matrix material. More specifically, the additional cost for the filler at this loading is about 1.5% of the materials cost, with is rather negligible, although additional preparation costs should be considered, too.

In the case of higher filled nanocomposites, as tested for the current study, nanoparticles could create agglomerations. Even slight agglomerations may not introduce processability difficulties in other AM techniques; in the case of the SLA process, the micro-sized laser spot and the locally introduced light energy for the polymerization procedure may cause serious damage to the 3D printing quality. Cuprous Oxide is a metal oxide that is vulnerable to agglomeration effects. Even though the agglomerations conducted on high-concentration nanocomposites were of low intensity, the studied SLA technology was critically affected. The degraded performance over 0.5 wt.% filled nanocomposites is plausibly attributed to such effects. Diffusion in the local range of agglomeration areas has forced the matrix resin material to not fully polymerize, degrading the total fusion of the nanocomposite.

The SLA process requires extra photopolymerization processing after washing in IPA dilution, which enhances the properties of the 3D printed material. Low photopolymerized nanocomposites with higher Cuprous Oxide loadings did not have the opportunity to upcycle during the curing process. This was a result of the washing process, during which uncured areas around the nanoparticles were removed from the IPA bath. The processing followed during the current study, the utilized materials, and the corresponding equipment used, provided the ability to fabricate nanocomposites with enhanced mechanical, thermal, and antibacterial performance. Additionally, threshold points were plausibly obtained after a comparative analysis of sufficient filler loadings. The addition of Cuprous Oxide nanoparticles in low concentrations, such as these of 0.5 wt.%, enabled the photosensitive SLA resin and enhanced its mechanical performance. Antibacterial performance was also sufficient in the nanocomposites, while higher Cuprous Oxide ratios could create even improved inhibition zones. An optimization study could be conducted as future work for the 3D printing settings to filler loadings to achieve the highest possible antibacterial performance without compromising the photopolymerization procedure, resulting in excellent mechanical behavior.

## 5. Conclusions

For the current study, a rather simple fabrication method was used to prepare nanocomposite materials with bacteria inhibiting properties for the SLA 3D printing process. More specifically, the effect of Cuprous Oxide addition in SLA-utilized resin matrix materials was investigated for the study. A common, rather low-cost, commercially available resin was utilized as a matrix material, and Cuprous Oxide nanoparticles were induced at different filling ratios. SLA 3D printing technology was utilized for the fabrication of the specimens, and international standards were followed for the mechanical, morphological, thermal, and antibacterial properties investigation on the prepared nanocomposites. The results indicated that the addition of filler at low ratios, such as these of 0.5 wt.%, could provide a significant advantage to SLA resins for implementation in engineering and medical applications. The antibacterial performance of the prepared nanocomposites was verified with the agar well diffusion method, while, at the same time, the introduction of the filler enhanced the mechanical response of the materials when compared with the matrix material of the study. These results revealed the high potential of these materials as multi-functional materials in applications requiring the inhibition of bacteria populations, which is of great interest in medicine and other fields [61].

The processing of such nanocomposites does not require procedures of high complexity, while the optimization of 3D printing settings consists of easy steps. Generally, the addition of Cuprous Oxide at concentrations higher than 0.5 wt.% ratios introduced slight agglomerations and plausibly low-quality polymerization. In contrast, antibacterial properties, which are significant for medical applications, exhibited an increased rate following the increased presence of Cuprous Oxide in each nanocomposite. Finally, this enables the potential of future studies for optimizing the process of the introduction of such nanoparticles in resin matrices to achieve optimum antibacterial performance in combination with ease of processing and enhanced mechanical behavior.

## Data Availability

The data presented in this study are available upon request from the corresponding author.
